# Hypoxia-induced hyperpermeability of rat glomerular endothelial cells involves HIF-2α mediated changes in the expression of occludin and ZO-1

**DOI:** 10.1590/1414-431X20186201

**Published:** 2018-05-17

**Authors:** Peng-Li Luo, Yan-Jun Wang, Yan-Yan Yang, Jia-Jia Yang

**Affiliations:** Department of Nephrology, Affiliated Hospital of Qinghai University, Xining, China

**Keywords:** Hypoxia, HIF-2α, Rat glomerular endothelial cells, Tight junction, Permeability

## Abstract

This study aimed to investigate the role of hypoxia-inducible factor-2α (HIF-2α) in the expression of tight junction proteins and permeability alterations in rat glomerular endothelial cells (rGENCs) under hypoxia conditions. The expression level of HIF-2α and tight junction proteins (occludin and ZO-1) in rGENCs were examined following 5% oxygen density exposure at different treatment times. HIF-2α lentivirus transfection was used to knockdown HIF-2α expression. Cells were divided into four groups: 1) control group (rGENCs were cultured under normal oxygen conditions), 2) hypoxia group (rGENCs were cultured under hypoxic conditions), 3) negative control group (rGENCs were infected with HIF-2α lentivirus negative control vectors and cultured under hypoxic conditions), and 4) Len group (rGENCs were transfected with HIF-2α lentivirus and cultured under hypoxic conditions). The hypoxia, negative control, and Len groups were kept in a hypoxic chamber (5% O_2_, 5% CO_2_, and 90% N_2_) for 24 h and the total content of occludin and ZO-1, and the permeability of rGENCs were assessed. With increasing hypoxia time, the expression of HIF-2α gradually increased, while the expression of occludin decreased, with a significant difference between groups. ZO-1 expression gradually decreased under hypoxia conditions, but the difference between the 24 and 48 h groups was not significant. The permeability of cells increased following 24-h exposure to hypoxia compared to the control group (P<0.01). The knockdown of HIF-2α expression significantly increased occludin and ZO-1 content compared with hypoxia and negative control groups (P<0.01), while permeability was reduced (P<0.01). Hypoxia increased HIF-2α content, inducing permeability of rGENCs through the reduced expression of occludin and ZO-1.

## Introduction

The kidney is sensitive to changes in oxygen delivery. Hence, this makes the kidney prone to hypoxia injury. Proteinuria studies have demonstrated the existence of a correlation with high altitude and hypoxia. For example, acute hypoxia causes a 2 to 3-fold increase in urinary protein excretion (1). Chronic hypoxia also increases the excretion of proteinuria. Five percent of Tibetans were found to have microalbuminuria (2) and 6/27 (22%) of chronic mountain sickness patients had proteinuria >1 g/24 h, which occurs to natives and long-time residents of altitudes above 2500 m because of hypoxia (3). The mechanism of hypoxia-induced proteinuria remains unclear. From a pathophysiologic point of view, the presence of protein in the urine reflects a size-selective dysfunction of the glomerular barrier and is often associated with hemodynamic, hypertension, diabetes, or glomerulopathy.

The glomerular barrier comprises the single glomerular capillary lined by the glomerular endothelial cells, the glomerular basement membrane, and the specialized epithelial cells, that is, podocytes that cover the basement membrane on the side facing the urinary space. The barrier allows for high filtration rates of water, non-restricted passage of small and middle-sized molecules, and almost total restriction of serum albumin and larger proteins. Perturbation of the components of the barrier can result in the clinical endpoints of proteinuria.

However, the molecular mechanisms that lead to proteinuria are poorly understood. One possibility is that it is due to abnormal endothelial function. There is now considerable evidence to suggest that contraction of endothelial cells may change intercellular cleft size by endothelial tight junction modulation (4), and that transcellular holes influence fluid and macromolecular movement across the vascular wall (5).

The tight junction (TJ) between cells is important for maintaining capillary permeability. The intercellular gap is increased when the TJ is damaged by various causes, which may lead to the increase in vascular permeability (6). The disruption of the TJ between glomerular endothelial cells (GENCs) may induce capillary hyperpermeability, proteinuria, inflammatory cell infiltration, and progression of kidney disease (7,8). However, little is known about the modulation of TJ and the mechanisms underlying these changes in a hypoxia environment.

Hypoxia-inducible factor (HIF), a basic helix-loop-helix transcription factor composed of an oxygen-sensitive α subunit and a constitutively expressed β subunit, is an important regulatory factor that allows individual cells to adapt to hypoxia (9). Under normoxic conditions, the HIF-α is hydroxylated by specific prolyl hydroxylase (PHD) and is rapidly degraded via the ubiquitin-proteasomal system. In hypoxia, PHD-mediated hydroxylation is inhibited, and HIF-α escapes degradation and dimerizes with HIF-β to drive the transcription of target genes that control a variety of adaptive responses to hypoxia (10,11). HIF-1α is expressed weakly in the outer cortex and strongly in some tubular and collecting duct epithelial cells. HIF-2α was localized to glomeruli with dense staining in the nuclei of podocytes and microvascular endothelial cells (9). In one report, mice with HIF-2α deficiency in endothelial cells presented increased vessel permeability, although without involvement of tight junction proteins (12). This indicates that HIF-2α may interfere in normal permeability of blood vessels, although the precise mechanisms in each different condition remain unclear.

To further investigate the mechanisms of cell hyperpermeability induced by hypoxia, we analyzed the effects of hypoxia on TJ proteins in rat glomerular endothelial cells (rGENCs), as well as the role of HIF-2α in the underlying mechanism of barrier regulation.

## Material and Methods

### Chemicals and antibodies

Antibodies to HIF-2α, occludin, and zonula occludens (ZO-1) were purchased from Invitrogen Life Technologies (USA) and GAPDH was purchased from Proteintech Group Inc. (USA). Millicell-ERS was purchased from Millipore (USA). Lipofectamine 2000, 293T cells and Opti-MEM I reduced Serum Media were purchased from Invitrogen.

### Cell culture

rGENCs were purchased from ATCC (USA). rGENCs cultures were established and characterized, as previously described (13). Briefly, rGENCs were grown in RPMI-1640 medium (Gibco-BRL, USA) supplemented with 10% fetal bovine serum (Gibco-BRL) and 10% NuSerum (Sigma-Aldrich, USA) in a cell incubator at 37°C under 5% CO_2_. When cells reached 70–80% confluence, the cultures were transferred into an automatically controlled Multi Gas Incubator (YCPHOSPHO- 50S, BaiDianTech, China), in which the oxygen levels (5% O_2_, 5% CO_2_, and 90% N_2_) and temperature (37°C) were maintained for various incubation durations (6, 12, 24, and 48 h). The control group was exposed to a normal environment (20.9% O_2_). Then, HIF-2α lentivirus transfection was used to knock down HIF-2α expression in rGENCs. These cells were divided into four groups: 1) control group (rGENCs were cultured under normal oxygen conditions); 2) hypoxia group (rGENCs were cultured under hypoxic conditions); 3) negative control group (rGENCs were infected with HIF-2α lentivirus negative control vectors and cultured under hypoxic conditions); and 4) Len group (rGENCs were transfected with HIF-2α lentivirus and cultured under hypoxic conditions). The hypoxia, negative control, and Len control groups were kept in a hypoxic chamber (5% O_2_, 5% CO_2_, and 90% N_2_) for 24 h, then the total content of occludin and ZO-1 and the permeability of rGENCs were assessed.

### Lentiviral vector construction and transfection

The lentivirus vector system and plasmids (pLP1/pLP2/VSVG) used to silence HIF-2α expression in rGENCs were purchased from Shanghai BIOSH Company (China). One shRNA sequence target of mRNAHIF-2α (*Rattus norvegicus*, NM-023090.1) from 1237 to 1257 bp gene was synthesized, annealed, and ligated into the pGMLV-SC5 vector with BamH I/EcoR I sites. A scrambled shRNA was used as a negative control. The recombinant lentivirus was packaged by transfecting the shRNA plasmids and lentiviral packaging vectors into 293T cells, according to manufacturer’s instructions of Lipofectamine 2000 (Invitrogen). At 48 h after transfection, lentivirus particles were harvested from the culture medium. rGENCs were seeded into 6-well plates and cultured for 24 h, the supernatant was discarded and 200 µL/well of virus suspension was added to medium containing polybrene (concentration is 5 µg/mL). Following 12 h of cell culture, puromycin (0.5 µg/mL) was added to the medium and stable clones were maintained in 1 μg/mL puromycin until distinct colonies appeared large enough for colony picking. The colony cells were selected and cultured in culture bottle with DMEM containing 10% FBS for subsequent experiments. Knockdown efficiency was determined by western blot analysis after infection.

### Western blot analysis

Endothelial cells were treated with lysis buffer and centrifuged at 15.000 *g* for 10 min at 4°C. The supernatant was collected for western blotting or stored at -80°C. Equal quantities of protein were loaded onto a gel for 15% SDS-PAGE (Sigma-Aldrich). Separated proteins were transferred onto polyvinylidene difluoride membranes (Millipore, France) and incubated with rabbit anti-rat ZO-1 and occludin antibody (1:2.000; Santa Cruz Biotechnology, Inc. USA) overnight at 4°C. After washing with phosphate-buffered saline (PBS), an appropriate horseradish peroxidase-conjugated goat anti-rabbit polyclonal secondary antibody (1:3.000; Beyotime Inc., China) was added and cultured for one hour at room temperature. Band intensities were quantified by Quantity One 2.0 software (Bio-Rad, USA). The experiment was repeated 3 times.

### Transendothelial electrical resistance (TEER)

The electrical resistance across the confluent cell monolayer was measured using the Millicell-ERS system (Millipore), according to manufacturer’s instructions. Briefly, cells were grown to post-confluence on transwell filters (Corning Costar Inc., USA) and treated according to protocol. The shorter electrode was placed within the Millicell culture plate insert and the longer electrode was placed in the outer well. The resistance of the culture or cell-free plate (blank) was measured from three different equidistant points across the inner and outer wells until a stable value was measured each time. The resistance of the blank was subtracted from that measured with endothelial cells (net resistance). The TEER unit area (KΩ·cm^2^) was calculated by multiplying the net resistance by the area of the culture plate insert.

### Statistical analysis

All experiments were repeated at least three times. Data are reported as means±SD. Statistical analysis was carried out using SPSS 16.0 software (SPSS Inc., USA). One-way ANOVA was used to detect the differences among groups. P<0.05 was considered statistically significant.

## Results

### Efficiency assessment of lentiviral vector transfection in rGENCs

**Figure 1.**
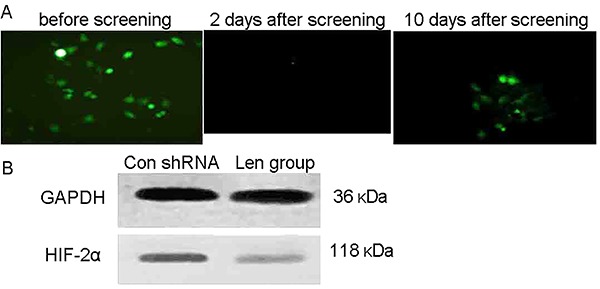
Efficiency assessment of lentiviral vector transfecting in rat glomerular endothelial cells (rGENCs). *A,* Screening of monoclonal cells by puromycin amino nucleoside. The distinct colonies were formed at 10 days after screening by puromycin (fluorescence microscopy ×200). *B,* Expression of HIF-2α was detected by western blot to evaluate the efficiency of lentiviral vector transfecting in rGENCs. Con shRNA: negative control group; Len group: HIF-2α lentivirus transfected group; GAPDH: glyceraldehyde-3phosphate dehydrogenase.

Puromycin was used on the selected monoclonal cell population after lentiviral particle transfection. Cell proliferation and survival could be observed through the display of enhanced green fluorescent particles using a fluorescent microscope. The distinct colonies were formed at 10 days after screening by puromycin. The expression of HIF-2α was detected by western blot, and results confirmed that the expression level of HIF-2α was restrained (Figure 1). This indicated that the transfection condition was set stably. Cells were successfully transfected and were stored for follow-up experiments.

### Influence of hypoxia on the expression of HIF-2α, occludin, and ZO-1

**Figure 2.**
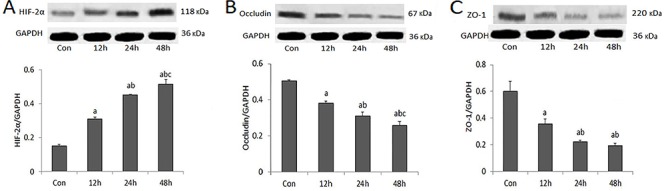
Influence of hypoxia on the expression of HIF-2α, occludin, and ZO-1. *A,* with the elongation of hypoxia time, the expression of HIF-2α gradually increased and *B,* occludin gradually decreased. *C,* Expression of ZO-1 also gradually decreased under hypoxia conditions, the differences being statistically significant, except for comparison between 24 and 48 h. Data are reported as means±SD. ^a^P<0.01 compared with control group; ^b^P<0.01 compared with 12 h; ^c^P<0.01 compared with 24 h (ANOVA). Con: control group.

In order to examine the effect of treatment duration, cells were exposed to hypoxia for 12, 24, and 48 hours. Results revealed that the expression of HIF-2α was significantly higher (P<0.01), and the expression of occludin and ZO-1 significantly lower (P<0.01) compared to the control group. With the elongation of hypoxia time, the expression of HIF-2α gradually increased (Figure 2A) while the expression of occludin (Figure 2B) decreased. The expression of ZO-1 also gradually decreased under hypoxia conditions, the differences being statistically significant, except for comparison between 24 and 48 h (Figure 2C).

### Influence of HIF-2α on the expression of occludin and ZO-1

**Figure 3.**
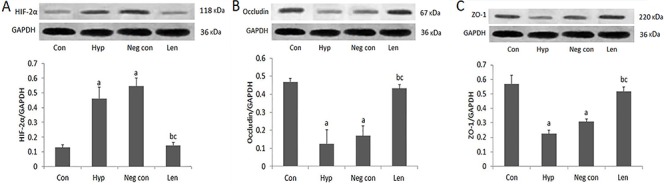
*A,* Hypoxia led to HIF-2α increase in cells, except in cells transfected with HIF-2α-shRNA. *B,* After infection with HIF-2α-shRNA, the expression of occludin significantly increased in rat glomerular endothelial cells (rGENCs). *C*, The expression of ZO-1 under hypoxia conditions also increased. Data are reported as means±SD. Con: control group, rGENCs were cultured under 20.9% O^2^ (n=3); Hyp: hypoxia group, rGENCs were cultured under 5% O^2^ (n=3); Neg con: negative control group, rGENCs were infected with HIF-2α lentivirus negative control vectors and cultured under hypoxic conditions (n=3); Len: HIF-2α lentivirus transfected group, rGENCs were transfected with HIF-2α lentivirus and cultured under hypoxic conditions (n=3). ^a^P<0.01 compared with control group; ^b^P<0.01 compared with hypoxia group; ^c^P<0.01 compared with Neg con group (ANOVA).

In order to explore the role of HIF-2α in TJ protein, HIF-2α-shRNA lentivirus was allowed to infect rGENCs, which showed lower HIF-2α expression levels. Non-silencing shRNA lentivirus was produced to infect rGENCs as the negative control group. The results showed that hypoxia led to HIF-2α increase in cells, except in cells transfected with HIF-2α-shRNA (Figure 3A). After infection with HIF-2α-shRNA, the expression of occludin increased significantly in rGENCs under hypoxia conditions, compared to the hypoxia and negative control groups (Figure 3B, P<0.01). The data are reported in Table 1. The expression of ZO-1 under hypoxia conditions also increased in rGENCs after infection with HIF-2α-shRNA, compared to the hypoxia and negative control groups (Figure 3C, P<0.01)

**Table 1.** Expression of HIF-2α, occludin, and ZO-1 in different groups.

Group	Con	Hyp group	Neg con group	Len group
HIF-2α	0.13±0.02	0.46±0.08^a^	0.54±0.06^a^	0.14±0.02^bc^
Occludin	0.47±0.04	0.13±0.02^a^	0.17±0.02^a^	0.43±0.04^bc^
ZO-1	0.57±0.06	0.22±0.03^a^	0.31±0.02^a^	0.52±0.03^bc^

Data are reported as means±SD. Con: control group; Hyp: hypoxia group; Neg con: Negative control group; Len: HIF-2α lentivirus transfected group. ^a^P<0.01 compared with control group; ^b^P<0.01 compared with hypoxia group; ^c^P<0.01 compared with Neg con group (ANOVA).

### Influence of HIF-2α on the permeability of rGENCs

**Figure 4.**
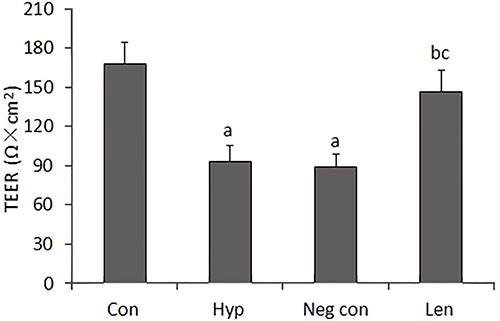
Influence of HIF-2α on the permeability of rat glomerular endothelial cells. Data are reported as means±SD (ANOVA). Con: control group; Hyp: hypoxia group; Neg con: Negative control group; Len: HIF-2α lentivirus transfected group. ^a^P<0.01 compared with control group; ^b^P<0.01 compared with hypoxia group; ^c^P<0.01 compared with Neg con group (ANOVA).

After 24 h of exposure to hypoxia, rGENCs exhibited a significant decrease in TEER compared to the control group (P<0.01). This indicated that the permeability of rGENCs was elevated after exposure to hypoxia. After infection with HIF-2α-shRNA, TEER became significantly higher compared to the hypoxia and negative control groups under hypoxia conditions (P<0.01). The difference between the hypoxia and negative control groups was not significant (P>0.05) (Figure 4).

## Discussion

Hypoxia is one of the most common causes of vascular hyperpermeability (14–16). Following hypoxic exposure, the vascular permeability of the brain revealed a two-fold increase in fluorescence intensity, which is indicative of significant vascular leakage (17). Some studies have found that the integrity of the blood-brain barrier in rats is disrupted and permeability increased in hypoxic conditions, and the TJ proteins occludin and ZO-1 downregulation or derangement may be responsible for these changes (18,19).

TJ is the most apical structure within the intercellular cleft, creating a paracellular barrier that is essential for survival of complex organisms. Integral TJ proteins are linked to each other as well as to the cytoskeleton by cytoplasmic adaptor proteins, such as ZO and occludin that provide the material foundation to restrict vascular permeability to molecules, allowing them to either diffuse across cell membranes or be carried across the membranes by specific membrane transporters (20–22). TJ proteins are dysregulated or can be genetically defective in numerous diseases, which may lead to three effects: i) impaired paracellular transport causing magnesium loss in the kidney, ii) increased paracellular transport of solutes and water causing leak-flux diarrhea, and iii) increased permeability to large molecules (23). A large body of evidence suggests that occludin and ZO-1 are the major components of endothelial TJ; changes in the localization, expression or phosphorylation of occludin/ZO-1 can lead to changes in TJ dysfunction and contribute to hyperpermeability. The rat brain endothelial cell line RBE4 exposure to hypoxia rapidly induced TJ disruption mainly through delocalization and increased tyrosine phosphorylation of occludin and ZO-1 with blood-brain barrier impairment (24). Chao et al. reported that high glucose (30 mM) significantly increased paracellular permeability and attenuated expression of ZO-1 and occludin in HUVECs by enhancing amyloid precursor protein expression with increased amyloid beta-peptide production (25). This suggests that TJ proteins, such as occluding and ZO-1, play an important role in maintaining ideal vascular permeability and this regulation may be affected by hypoxia. In this study, we found that the expression of occludin and ZO-1 gradually decreased with the elongation of hypoxia time, meanwhile cell permeability was increased after 24-h exposure to hypoxia, both being significantly different compared to the control group.

This suggests that the hyperpermeability of cells under hypoxic conditions may be related to the reduction in occludin and ZO-1. A variety of occludin and ZO-1 expression patterns can occur under hypoxia conditions, such as the reduction of both in endothelial cells (19,26,27), no expression changes in bone marrow (28) and brain endothelial cells after treatment for six hours by hypoxia (29), or a significant reduction in occludin expression, and no change in ZO-1 expression levels (30). It was speculated that experimental environment (*in vivo* or *in vitro*), culturing conditions, cell type, exposure time, and stimulus conditions are involved in this process. However, the protein occludin plays a crucial role in keeping the normal function of TJ and its downregulation is enough to cause the dysfunction of TJ (31,32).

Hypoxia-inducible factors (HIFs) are ubiquitous master regulators of such hypoxic adaptation. HIF is a heterodimer composed of α-subunit and β-subunit. HIF-α is regulated by oxygen-dependent proteolysis. Most cell types express HIF-1α, while HIF-2α shows a more restricted pattern of expression. In the kidney, HIF-1α is expressed in tubules, while HIF-2α is confined to endothelial and interstitial cells (33). HIF-2α was localized in glomeruli endothelial cells and podocytes in kidneys (9). Mice with HIF-2α deficiency in endothelial cells developed normally, but displayed a variety of phenotypes, including increased vessel permeability and aberrant endothelial cell ultrastructure (12). Some studies indicated that endothelial HIF-2α play a protective role against ischemia of the kidney. In a previous study, mice were more susceptible to renal ischemia-reperfusion injury such as elevated blood urea nitrogen when HIF-2α expression was approximately one-half that of wild-type mice, whereas HIF-1α expression was equivalent (28). Inactivation of endothelial HIF-2α, but not endothelial HIF-1α, resulted in increased expression of renal injury markers and inflammatory cell infiltration in the post-ischemic kidney (34). Those results indicated that HIF-2α plays an important role in maintaining the normal permeability of blood vessels and protecting from ischemic renal damage. In our study, we found that the expression of HIF-2α increased gradually with the elongation of hypoxia time while occludin and ZO-1 expression decreased with the hyperpermeability of cells. After knockdown of HIF-2α expression, the content of occludin and ZO-1 was upregulated, and the permeability of cells decreased at the same time. Therefore, we hypothesized that HIF-2α may have affected the permeability of endothelial cells through the regulation of occludin and ZO-1.

In conclusion, our results suggest that hypoxia could promote the increase of HIF-2α content, which could induce increased permeability of rGENCs through reduction of the expression of occludin and ZO-1.
